# Evaluation of Nutritional Status of Patients with Depression

**DOI:** 10.1155/2015/521481

**Published:** 2015-08-27

**Authors:** Gülşah Kaner, Meltem Soylu, Nimet Yüksel, Neriman Inanç, Dilek Ongan, Eda Başmısırlı

**Affiliations:** ^1^Department of Nutrition and Dietetics, Faculty of Health Sciences, Nuh Naci Yazgan University, 38170 Kayseri, Turkey; ^2^Kayseri Education and Research Hospital, 38170 Kayseri, Turkey; ^3^Department of Nutrition and Dietetics, Faculty of Health Sciences, Izmir Kâtip Çelebi University, 35620 Izmir, Turkey

## Abstract

*Aims and Objectives*. Our goal was to determine nutritional status, body composition, and biochemical parameters of patients diagnosed with depression based on DSM-IV-TR criteria.* Methods*. A total of 59 individuals, aged 18–60 years admitted to Mental Health Centre of Kayseri Education and Research Hospital, were included in the study. The participants were randomly assigned to two groups; depression group (*n* = 29) and control group (*n* = 30). Anthropometric measurements, some biochemical parameters, demographic data, and 24-hour dietary recall were evaluated.* Results*. 65.5% of depression and 60.0% of control group were female. Intake of vitamins A, thiamine, riboflavin, B6, folate, C, Na, K, Mg, Ca, P, Fe, Zn, and fibre (*p* < 0.05) were lower in depression group. Median levels of body weight, waist circumference, hip circumference, waist-to-hip ratio (*p* < 0.05) were significantly higher in depression group. Fasting blood glucose levels, serum vitamins B12, and folic acid (*p* < 0.05) in depression group were lower than controls. Serum insulin and HOMA levels of two groups were similar.* Conclusion*. Some vitamin B consumption and serum vitamin B12 and folic acid levels were low while signs of abdominal obesity were high among patients with depression. Future research exploring nutritional status of individuals with depression is warranted.

## 1. Introduction

Depression alone accounts for 4.3% of the global burden of disease and is among the largest single causes of disability worldwide (11% of all years lived with disability globally), particularly for women [1]. Studies about associations between diet and depression have primarily focused on single nutrients or foods. Recently, dietary patterns representing a combination of foods have attracted more interest than an individual nutrient. Depression is a serious effective illness with a high lifetime prevalence rate in which diet has been suggested as one modifiable factor [2].

An association between diet and depression has now been confirmed in prospective and epidemiological studies. For example, in elderly men and women, consumption of fish, vegetables, olive oil, and cereals was negatively correlated with severity of depressive symptoms [3]. The benefits from fish and olive oil intake remained significant even when adjusted for confounders such as age, sex, educational status, BMI, and physical activity level as well as the presence of a number of medical conditions [4]. In a prospective study, after adjusting for sex, age, smoking status, BMI, physical activity levels, and employment status, adherence to a Mediterranean diet including high levels of vegetables, fruits, nuts, cereals, legumes, and fish, moderate alcohol intake, and low amount of meat or meat products and whole-fat dairy intake was protective against development of depression [4]. In a study by Jacka et al. [5], consuming a “traditional” diet containing vegetables, fruits, meat, fish, and whole grains was also associated with a 35% reduced risk of depression or dysthymia. Researches about diet of adolescents [5] and of the community-dwelling elderly with low socioeconomic level [6] have also provided evidence for an association between diet quality and depression. Depressive symptoms are also positively associated with consumption of sweets [7]. Similarly, high consumption of fast food and processed pastries is associated with an increased risk for depression up to 6 years later [8]. In a randomised-controlled trial, six days on a low protein diet significantly decreased depressive symptoms in patients with type 2 diabetes mellitus [9] and, in another randomised study about overweight and obese individuals, those who were placed on an energy-restricted, low-fat diet for one-year experienced greater improvements in mood compared to participants on an energy-restricted, low-carbohydrate diet [10]. These changes were independent of weight loss. PUFAs, particularly omega-3 essential fatty acids (EFA), have received significant attention in relation to depression. In a meta-analysis of 14 studies comparing the levels of PUFAs between depressed patients and control subjects, levels of eicosapentaenoic acid (EPA), docosahexaenoic acid (DHA), and total n-3 EFA were significantly lower in depressed patients than controls. There was no significant change in arachidonic acid (AA) or total n-6 PUFAs [11]. A meta-analysis of 15 clinical trials about effects of EPA supplementation in depressed populations revealed beneficial effects from fish oil containing high levels of EPA [12]. Other investigations about relationship between nutrients and depression have also demonstrated a role of folate [13, 14], Zn [15–17], Fe [18, 19], vitamin B_6_ [20–22], and vitamin B_12_ [21, 23].

Obesity is associated with an increased risk of mental illness however; evidence linking BMI to mental illness is inconsistent [24]. Whether obesity also predicts psychiatric disorders such as depression has not been established. Thus far, population-based studies of association between obesity and depression have yielded inconsistent results [25]. Some studies found an association [26–28], while others did not [29–31]. Depressive symptoms may contribute to abdominal obesity through consumption of diets with high energy density [32]. Grossniklaus et al. [32] have determined that depressive symptoms and dietary energy density were associated with elevated waist circumference. Among overweight and obese U.S. adults, high waist circumference or abdominal obesity was significantly associated with increased likelihoods of having major depressive symptoms or moderate-to-severe depressive symptoms. Zhao et al. [33] stated that mental health status should be monitored and evaluated in adults with abdominal obesity, particularly in those who are overweight.

These results suggest that healthy dietary pattern is significantly associated with major depression in adults. Further studies are needed to confirm them, however. In addition, to the best of our knowledge, there is no study of its kind in our country to evaluate nutritional intake of patients with depression. Therefore, the present study was conducted to determine nutritional status, body composition, and selected biochemical parameters of patients diagnosed with depression based on DSM-IV-TR criteria and to compare their data those of a control group.

## 2. Methods

This randomized controlled study was performed at our Mental Health Centre Clinic of Kayseri Education and Research Hospital of Medicine, a tertiary referral centre in Turkey.

Forty-two inpatients diagnosed with major depression in accordance with Diagnostic and Statistical Manual of Mental Disorders, Fourth Edition (DSM-IV-TR), who are on antidepressant medication and thirty-two normal healthy volunteers aged between 18 and 60 were studied between 2009 and 2010. Of 74 subjects, 13 patients and 2 healthy subjects had missing information thus data of 59 participants (29 patients in depression group and 30 healthy volunteers in controls) were used.

Demographic characteristics like age, gender, family status (married, divorced, and widowed), financial status (average annual income during the past three years), smoking habits, alcohol consumption, and occupational status as well as education level were obtained with questionnaire by face-to-face interview. There were no differences between groups across race/ethnicity.

Food consumption frequency, energy, and nutrient intake by 24-hour dietary recall were determined. Daily energy expenditure and physical activity levels were calculated. Height, body weight, waist circumference, hip circumference, BMI, and waist-to-hip ratio were also determined. Selected biochemical parameters were evaluated (fasting blood glucose, folate, vitamin B_12_, and insulin).

Exclusion criteria were derived as follows: (1) serious mental illness (e.g., a psychotic disorder, bipolar disorder, posttraumatic stress disorder, schizophrenia, anxiety disorders, dementia, or bulimia), (2) recent initiation or dose adjustment of thyroid medications, and (3) weight loss medication or treatment, bariatric surgery, diabetes, and pregnancy.

All participants provided written informed consent, and the protocol was approved by the institutional review boards of the participating centers, in accordance with the Declaration of Helsinki. The institutional review board of the Ethics Committee of Faculty of Medicine in Erciyes University (Kayseri, Turkey) approved the study protocol on November 5, 2009. This trial was registered with number 2009/130.

### 2.1. Data Collection

#### 2.1.1. Preparation and Implementation of the Questionnaire

The questionnaire was designed in a manner understandable for individuals and was based on literature review [6, 34, 35]. Comprehensibility of questions in the questionnaire was tested on 20 individuals, necessary adjustment was made accordingly, and questionnaire was finalized. The questionnaire included an overview of individual's eating habits, anthropometric measurements, food consumption frequency, energy and nutrient intake, and physical activity level with 24-hour recall. The questionnaire took approximately 45 minutes to administer for each participant.

#### 2.1.2. Anthropometric Measurements

Anthropometric measurements were determined according to WHO criteria [36]. Body weight, height, waist, and hip circumferences were measured and BMI was calculated (BMI = body weight (kg)/height (m^2^)). All subjects were weighed while wearing light clothing and being without shoes, using a calibrated digital flat scale (Seca-803, USA). Standing height was measured without shoes to the nearest 0.5 cm with a measuring tape. All anthropometric measurements were measured three times and mean values were obtained. BMI values were evaluated using WHO classification which shows that a BMI less than 20.0 kg/m^2^ is classified as underweight, between 20.0 and 24.9 kg/m^2^ is defined as normal weight, between 25.0 and 29.9 kg/m^2^ as overweight, between 30.0 and 34.9 kg/m^2^ as 1st-degree obesity, and between 35.0 and 39.9 kg/m^2^ as 2nd-degree obesity, and BMI higher than 40 kg/m^2^ is classified as 3rd-degree (morbid) obesity [37].

#### 2.1.3. Assessment of Food Consumption

Nutritional behaviour of participants was determined by food consumption frequency and 24-hour dietary recall. Nutrient Database (BeBiS, Ebispro for Windows, Germany; Turkish Version/BeBiS 7) was used to determine energy and nutrient intake; results were compared to Dietary Guidelines for Turkey [38]. Lower than 67% of recommended daily intake of energy and nutrients was evaluated as inadequate. Volumes and portion sizes were estimated with 2-dimensional food models and with a portion size picture booklet including 120 photographs of foods, each with 3–5 different portion sizes [38, 39].

#### 2.1.4. Assessment of Energy Expenditure

The participants recorded their activity level over a 24-hour period. Closed attention was given that activity duration equaled 24 hours. To determine energy expenditure per activity type, physical activity level (PAL) was calculated. PAL and basal metabolic rate (BMR) were multiplied and daily energy expenditure was obtained [37]. BMR was calculated according to the following formulas based on age and gender [37]:

 For men aged 19–30 years, 15.3 × body weight (kg) + 679 kcal.
 For women aged 19–30 years, 14.7 × body weight (kg) + 496 kcal.
 For men aged 31–60 years, 11.6 × body weight (kg) + 879 kcal.
 For women aged 31–60 years, 8.7 × body weight (kg) + 829 kcal.
 For men aged >60 years, 13.5 × body weight (kg) + 487 kcal.
 For women aged >60 years, 10.5 × body weight (kg) + 596 kcal.

### 2.2. Assessment of Biochemical Parameters

#### 2.2.1. Sample Collection and Preparation

Venous blood samples were collected after overnight fasting. Blood samples were incubated for one hour at room temperature; sera were separated and then stored at –20°C until biochemical analysis. Blood samples with anticoagulant were immediately centrifuged and plasmas were separated and stored at –70°C until insulin analysis. In patients, HOMA-IR (Homeostasis Assessment Model of Insulin Resistance) method (fasting insulin mU/mL × fasting glucose mmol/L/22.5) was used. In the HOMA-IR test, a minimum value of 2.5 was accepted as insulin resistance.

#### 2.2.2. Biochemical Analysis

Serum fasting blood glucose, triglyceride (TG), total cholesterol (C), HDL-C, and LDL-C concentrations were determined with kits by Architect c16000 autoanalyzer (Abbott Diagnostics, USA). Vitamin B_12_ and folic acid concentrations were determined with Advia Centaur XP immunoassay system (Siemens, Germany) with kits by Advia Centaur XP immunoassay system.

### 2.3. Statistical Analysis

Data were analysed with SPSS version 15.0 (Inc., Chicago, IL, USA). Normal distribution of data was determined with Shapiro-Wilk test. Chi-square analysis was used to compare the difference of qualitative variables between groups and Mann Whitney* U* test was used for quantitative data by showing median, 25%–75% percentages. *p* < 0.05 was set as statistically significant.

## 3. Results

Mean ages of depression (36.82 ± 1.86 years) and control (33.13 ± 1.57 years) groups were similar (*p* > 0.05). 65.5% of depression group and 60.0% of control group were females. Depression group that indicated that food consumption increased (20.7%) during times of sadness was significantly higher compared with controls (6.7%) (*p* < 0.05). Controls who indicated no changes in food consumption while experiencing nervousness (46.7%) or happiness (73.3%) were found to be significantly higher compared to the depression group (6.9% and 31.0%, resp., *p* < 0.05). Between-meals consumption of depression group (82.8%) was significantly lower than controls (100%, *p* < 0.05). Rate of night eating in depression group was 41.4% while it was 13.3% in controls (*p* < 0.05).

Ratio of daily fresh fruit consumption was lower in depression group (13.8%) than in controls (50.0%). Daily consumption of fresh vegetables was 31.1% in depression group while it was 46.7% in controls. Of depression group, 65.4% consumed fish which was significantly lower than controls (83.3%). Among depression group, 10.3% of individuals were sedentary. Light physical activity levels were higher in depression group (86.2%) compared with the controls (56.7%). A statistical significance was found among physical activity levels between groups (*χ* ^2^ = 14.819, *p* < 0.05). There was no difference between the groups in terms of smoking and alcohol consumption.

Although statistically insignificant, polyunsaturated fatty acids (PUFA) intake of controls [10.53 (8.29–13.91) g] was higher than of depression group [7.62 (5.82–12.49) g] (*z* = −1.933, *p* > 0.05). Intakes of vitamins A (*p* < 0.05), thiamine (*p* < 0.05), riboflavin (*p* < 0.05), vitamins B_6_ (*p* < 0.05), folate (*p* < 0.05), vitamin C (*p* < 0.05), Na (*p* < 0.05), K (*p* < 0.05), Mg (*p* < 0.05), Ca (*p* < 0.05), P (*p* < 0.05), Fe (*p* < 0.05), Zn (*p* < 0.05), and fibre (*p* < 0.05) were lower in depression group (Table 1). According to Dietary Guidelines for Turkey, intake of fibre, niacin, vitamins B_6_, C (*p* < 0.05 for each), and Mg (*p* < 0.05) was lower in women with depression while intake of energy, fibre, vitamins A, E, B_6_, and C (*p* < 0.05 for each), and folate (*p* < 0.05) were lower in men with depression.

Median levels of body weight (*p* < 0.05), waist circumference (*p* < 0.05), hip circumference (*p* < 0.05), and waist-to-hip ratio (*p* < 0.05) were higher in depression group (Table 2). 1st- and 2nd-degree obesity were higher in depression group (27.6% and 13.8%, resp.) compared to controls (6.7% and 0.0%, resp.) (*p* < 0.05, Table 3). Median daily energy expenditure of depression group [1946 kcal (1827–2188 kcal)] was lower than of controls [2180 kcal (1944–2470 kcal)] (*p* < 0.05).

Fasting blood glucose levels (*p* < 0.05) and serum vitamins B_12_ (*p* < 0.05) and folic acid (*p* < 0.05) in depression group were lower than controls. Serum insulin and HOMA levels were not significantly different between groups (*p* > 0.05). Blood lipid levels of both groups were also similar (*p* > 0.05, Table 4).

## 4. Discussion

To the best of our knowledge, this is the first study of its kind in Turkey to evaluate nutritional intake, nutritional status, and some biochemical parameters of patients with depression. Results from this study indicated that depressed individuals increase their food intake as a response to negative emotions. Similar to this finding, Konttinen et al. [40] investigated an association between emotional eating and depressive symptoms. Emotional eating was related to higher consumption of sweet foods. In addition, depressive symptoms were related to a lower consumption of vegetables/fruit. We found higher rates of eating at night among patients with depression like Gluck et al. [41].

In this study, similar to previous studies, depressed patients' 24-hour food intake has shown a poor quality diet with lower intake of fruits/vegetables [40, 42]. This association of low fruit/vegetables intake with depression also led to inadequate intake of fibre in this study which is important in healthy life maintenance and protection from diseases [43].

On the other hand, consumption of fish was significantly lower in the depression group compared to controls and these results were consistent with previous studies [44–50]. Fish is the richest source of n-3 PUFA and EPA which has been found to be effective in relieving depression [49, 51]. Nevertheless, total PUFA intake was not different between people with and without depression in Meyer et al.'s study [51]. Similar to Meyer et al. [51], PUFA intake in the present study was similar in depression and control groups.

Intake of a number of nutrients (thiamin, riboflavin, vitamin B_6_, folate, and Na, K, Mg, Ca, P, Fe, and Zn) was significantly lower in the depression group compared to controls. Vitamins C and A are thought to be effective in depression due to their roles in the oxidative processes [35, 52]. In this study, patients in the depression group had significantly lower vitamins C and A intake than controls and could not meet their requirements according to Dietary Guidelines for Turkey. Similar to this study, Oishi et al. [53] indicated negative association between depressive symptoms and carotene and vitamin C intakes.

Folate and vitamin B_12_ are necessary for normal functioning of nervous system. They are also required for single carbon metabolism responsible for synthesis and metabolism of serotonin and other neurotransmitters [35]. All B vitamins work as a cofactor of the key enzymes for neurotransmitter production and to control their balance [54]. Similar to the present study, Pellegrin et al. [55] reported a low level of folate intake in depressed patients. Furthermore, depressed patients consumed less thiamine, riboflavin, and vitamin B_6_ than controls which show the overall inadequate intake of B vitamins in this study. In the Coronary Health Improvement Project (CHIP), conducted to decrease depression by modifying selected daily nutrients from food, a decrease in depression was achieved by increasing pyridoxine. [56].

Magnesium deficiency is known to cause neuropathologies. Lack of Mg leads to depression because of neuron damage occurring as a result of not meeting the Mg requirement of neurons [57]. Magnesium intake of the depression group in the present study was significantly lower than controls which may be due to insufficient consumption of food resources of Mg such as red meat, oilseeds, and nuts.

Inadequate dietary Zn and Fe intake contribute to depressive symptoms [35, 58, 59]. It was found in the present study that depression group consumed significantly lower amounts of Fe and Zn compared with controls, which may have resulted from low consumption of oil seeds.

Biological factors in depression occurrence are electrolyte imbalances especially Na and K, neurophysiological changes, autonomous nervous system dysfunction, and neuroendocrinological disorders in gonads, thyroid, hypophysis, adrenal cortex, and hypothalamus [60]. The present study findings demonstrated that depressed patients consumed lower amounts of Na, K, Ca, and P than controls.

The majority of literature demonstrates high prevalence of depression in people with high BMI [61–66]. It is still not clear whether depression leads to obesity in response to changing appetite and medicines or obesity contributes to depressive disturbances. Consistent with the literature findings, median body weight (kg) and BMI (kg/m^2^) of the depression group were significantly higher than controls in our study. Waist and hip circumferences and waist-to-hip ratios which show body fat distribution are important because chronic diseases, symptoms, and low quality of life are affected [67]. In a study conducted with 3186 adult males and 3003 adult females, depressed participants were found to have higher waist circumferences [68]. Besides body weight and BMI, we have found that waist-hip circumferences and waist-to-hip ratios were higher in patients with depression compared to controls.

High incidence of folic acid deficiency has been shown in patients with depression [45, 69–71]. The present study demonstrated lower serum folic acid levels in the depression group compared to controls, which may have resulted from low dietary folate consumption of the depressed patients. Vitamin B_12_ deficiency independently stimulates tetrahydrobiopterin production, retards monoamine neurotransmitters, and may cause functional folate deficiency [72]. In one study, people with vitamin B_12_ deficiency were found to have 2.05 times the risk of depression [73]. However, another study failed to show a significant difference between mean serum vitamin B_12_ levels of the depression and control groups [69]. In addition to these conflicting findings, the depression group in our study was found to have significantly lower serum levels of vitamin B_12_ compared to controls.

Depression is a symptom of impaired blood glucose tolerance [54]. One study demonstrated that depression in women was significantly related to increased blood glucose levels [74]. Conversely, in the present study, the median fasting blood glucose levels of the depression group were significantly lower compared to controls; however the fasting blood glucose levels of both groups were in normal range.

Depression is associated with an increased risk of incident diabetes; insulin resistance is thought to be the underlying link between them. Nevertheless, only a few studies have explored the association between insulin resistance and depression, with contradictory results [75–77]. A weak and positive correlation has been reported between scores identifying depression and HOMA-IR score [76, 77]. It was determined in the present study that HOMA was insignificantly higher in the depression group compared to controls with no insulin resistance in either group.

In this study, there are some limitations, the first of which is our small sample size. Large-scale studies are needed on this issue in the future studies. Second, self-reported dietary intake data are likely inaccurate.

## 5. Conclusion

Patients with depression were found to consume a poor quality diet which is known to lead to depressive symptoms. Besides low intake of some B vitamins, serum levels of vitamin B_12_ and folic acid were low, and there were many signs of abdominal obesity in the depression group. Therefore, future research exploring the overall nutritional status of individuals with depression is warranted in order to assist in understanding and treatment of the condition and to promote healthy lifestyles that may help in depression management.

## Figures and Tables

**Table 1:** Energy and nutrients consumption of depression and control groups.

Energy and nutrients	Depression group (*n* = 29)	Control group (*n* = 30)	*Z*	*p*
	Median (25% p–75% p)	Median (25% p–75% p)		
Energy (kcal)	1535 (1322–1710)	1498 (1380–1784)	**−0.121**	**0.910**
CHO (g)	210.2 (162.5–239.9)	181.8 (163.5–220.1)	**−1.183**	**0.240**
CHO %	55.0 (48.5–61.0)	50.0 (45.0–56.3)	**−1.921**	**0.055**
Fat (g)	51.8 (40.5–62.4)	52.4 (43.3–62.1)	**−0.030**	**0.982**
Fat %	31.0 (26.0–34.5)	32.0 (25.0–36.0)	**−0.448**	**0.659**
Protein (g)	57.1 (44.2–65.6)	64.3 (53.2–73.7)	**−1.926**	**0.055**
Protein %	14.0 (12.5–16.5)	17.0 (14.0–21.3)	**−2.419**	**0.015^**∗**^**
Cholesterol (mg)	168.2 (120.4–246.7)	166.5 (108.8–255.6)	**−0.243**	**0.816**
PUFA (g)	7.62 (5.8–12.5)	10.53 (8.3–13.9)	**−1.933**	**0.053**
Vitamin A (*μ*g)	516.6 (467.7–683.5)	670.5 (449.6–1249.6)	**−2.017**	**0.044^**∗**^**
Vitamin E (mg)	7.8 (5.6–11.7)	9.2 (7.0–15.4)	**−1.956**	**0.051**
Thiamine (mg)	0.5 (0.4–0.7)	0.7 (0.6–0.9)	**−2.889**	**0.003^*∗∗*^**
Riboflavin (mg)	0.8 (0.7–1.0)	1.1 (0.9–1.3)	**−3.754**	**<0.001**
Niacin (mg)	8.6 (6.83–11.43)	10.8 (7.3–13.3)	**−1.433**	**0.154**
Vitamin B_6_ (mg)	0.8 (0.6–1.0)	1.2 (0.9–1.4)	**−3.662**	**<0.001**
Vitamin B_12_ (*μ*g)	2.4 (1.3–4.3)	2.5 (1.4–3.6)	**−0.190**	**0.854**
Folate (*μ*g)	193.4 (149.8–268.3)	261.8 (220.4–318.6)	**−3.214**	**0.001^*∗∗*^**
Vitamin C (mg)	45.1 (21.6–70.3)	97.1 (40.3–191.9)	**−3.214**	**0.001^*∗∗*^**
Na (mg)	2734.8 (2159.0–3329.6)	3289.0 (2663.5–3810.9)	**−1.971**	**0.049^**∗**^**
K (mg)	1353.9 (1083.6–1653.2)	2063.9 (1665.1–2545.4)	**−4.533**	**<0.001**
Mg (mg)	183.2 (146.6–205.7)	244.3 (184.3–305.2)	**−3.654**	**<0.001**
Ca (mg)	367.9 (295.8–508.6)	592.7 (457.4–814.5)	**−4.351**	**<0.001**
P (mg)	744.7 (572.0–880.2)	899.1 (766.7–1081.2)	**−3.108**	**0.002^*∗∗*^**
Fe (mg)	8.5 (7.2–10.6)	10.6 (9.2–12.7)	**−2.593**	**0.009^*∗∗*^**
Zn (mg)	8.3 (7.2–10.4)	10.1 (8.3–11.3)	**−2.320**	**0.020^**∗**^**
Fibre (g)	13.3 (9.7–19.6)	21.5 (15.7–24.8)	**−3.131**	**0.001^*∗∗*^**

Median (25%–75%) represents median, 25th percentile and 75th percentile.

^*∗*^ *p* < 0.05 and ^*∗∗*^ *p* < 0.01.

**Table 2:** Anthropometric measurements of depression and control groups.

Anthropometric measurements	Depression group	Control group	*Z*	*p*
	(*n* = 29)	(*n* = 30)		
	Median	Median		
	(25% p–75% p)	(25% p–75% p)		
Height (m)	1.63 (1.60–1.71)	1.63 (1.57–1.73)	**−0.083**	**0.937**
Body weight (kg)	75.1 (64.7–88.6)	66.8 (62.2–79.0)	**−2.229**	**0.025^**∗**^**
BMI (kg/m^2^)	28.96 (24.37–33.46)	25.06 (22.08–27.26)	**−2.699**	**0.006^**∗∗**^**
Waist circumference (cm)	91.00 (83.50–100.00)	78.50 (75.00–90.00)	**−3.679**	**<0.001**
Hip circumference (cm)	105.00 (100.00–115.00)	100.50 (93.75–93.75)	**−2.778**	**0.005^**∗∗**^**
Waist-to-hip ratio	0.85 (0.82–0.93)	0.78 (0.76–0.87)	**−2.593**	**0.009^**∗∗**^**

Median (25%–75%) represents median, 25th percentile and 75th percentile.

^*∗*^ *p* < 0.05 and ^*∗∗*^ *p* < 0.01.

**Table 3:** Evaluation of body weight according to body mass index.

Body mass index (kg/m^2^)	Depression group		Control group		Total	
	(*n* = 29)		(*n* = 30)		(*n* = 59)	
	*n*	%	*n*	%	*n*	%
Underweight (<20)	0	0.0	49	13.3	4	6.8
Normal (20.0–24.9)	9	31.0	11	36.7	20	33.9
Overweight (25.0–29.9)	8	27.6	13	43.3	21	35.6
1st-degree obese (30–34.9)	8	27.6	2	6.7	10	16.9
2nd-degree obese (35.0–39.9)	4	13.8	0	0.0	4	6.8
3rd-degree obese (>40)	—	—	—	—	—	—
Total	**29**	**100**	**30**	**100**	**59**	**100**

*χ* ^2^ = 12.977; *p* < 0.05.

**Table 4:** Evaluation of biochemical parameters of depression and control groups.

Biochemical parameters	Depression group	Control group	*Z*	*p*
	(*n* = 29)	(*n* = 30)		
	Median	Median		
	(25% p–75% p)	(25% p–75% p)		
Glucose (mg/dL)	78.0 (72.0–87.0)	86.0 (82.7–89.2)	−2.559	**0.010^**∗**^**
Triglyceride (mg/dL)	124.0 (83.5–168.0)	106.5 (63.0–146.7)	−1.251	**0.214**
Total cholesterol (mg/dL)	186.0 (165.5–211.5)	188.5 (166.2–218.2)	−0.159	**0.877**
HDL-C (mg/dL)	43.0 (36.5–54.0)	49.0 (41.0–62.0)	−1.867	**0.062**
LDL-C (mg/dL)	116.0 (97.5–133.0)	116.5 (94.5–145.25)	−0.091	**0.931**
Vitamin B_12_ (pg/mL)	254.0 (203.5–339.0)	324.5 (264.0–383.0)	−2.153	**0.031^**∗**^**
Folate (ng/mL)	5.12 (3.63–6.81)	8.68 (6.00–10.10)	−3.571	**<0.001**
Insulin (*μ*U/mL)	12.5 (6.47–33.72)	9.22 (5.55–13.27)	−1.358	**0.179**
HOMA	2.45 (1.15–8.51)	2.29 (1.11–3.10)	−0.977	**0.337**

^*∗*^ *p* < 0.05.

## Relevance to Clinical Practice

By investigating nutritional status and dietary intake of patients with depression and providing adequate-balanced nutrition, healthcare professionals may contribute to the therapy of patients in the clinic.
